# Characterization of IGF2R Molecular Expression in Canine Osteosarcoma as Part of a Novel Comparative Oncology Approach

**DOI:** 10.3390/ijms24031867

**Published:** 2023-01-18

**Authors:** Charles Boisclair, Ryan Dickinson, Sabeena Giri, Ekaterina Dadachova, Valerie MacDonald-Dickinson

**Affiliations:** 1 Department of Small Animal Clinical Sciences, Western College of Veterinary Medicine, University of Saskatchewan, Saskatoon, SK S7N 5B4, Canada; 2 Department of Veterinary Pathology, Western College of Veterinary Medicine, University of Saskatchewan, Saskatoon, SK S7N 5B4, Canada; 3 College of Pharmacy and Nutrition, University of Saskatchewan, Saskatoon, SK S7N 5E5, Canada

**Keywords:** osteosarcoma, canine, IGF2R, radioimmunotherapy, immunohistochemistry, comparative oncology

## Abstract

Progress in prognostic factors, treatments, and outcome for both canine and human osteosarcoma (OS) has been minimal over the last three decades. Surface overexpression of the cation independent mannose-6-phosphate/insulin-like growth factor receptor type 2 (IGF2R) has been proven to occur in human OS cells. Subsequently, radioimmunotherapy (RIT) targeting IGF2R has demonstrated promising preliminary results. The main aims of this study were to investigate the expression of IGF2R in spontaneously occurring canine OS cells using immunohistochemistry (IHC) on archived biopsy samples and to assess its prognostic significance. Thirty-four dogs were included in the study. All cases showed that 80–100% of OS cells stained positive for IGF2R. IGF2R overexpression alone was not shown to have prognostic significance using both visual and quantitative methods of IHC staining intensity. This study has established for the first time the consistent expression of IGF2R in spontaneously occurring canine OS. This comparative oncology approach will allow further investigation into RIT as a novel treatment modality; first in canines and then in humans with OS. In addition, further studies should be performed to assess the true prognostic significance of IGF2R overexpression.

## 1. Introduction

Osteosarcoma (OS) is the most common malignant primary bone tumor in dogs and humans, accounting for 85–98% of malignancies originating in the skeleton in dogs [1,2] and 55% in children and adolescents [3]. Canine OS carries a poor prognosis with approximately 90% of affected patients dying from pulmonary metastasis within 4 months following amputation alone [4]. Adjuvant chemotherapy, commonly carboplatin, results in improved median survival times (ST), varying from 9–12 months [5,6,7]. More recently, other therapies such as stereotactic radiation [8,9,10] and tyrosine kinase inhibitors [11] have been evaluated without improvement of overall survival. Similar to canines, the complex karyotype and substantial aneuploidy of this tumor has resulted in a plateau in the overall survival at approximately 70% in humans treated with conventional therapies [12,13,14]. Therefore, new therapeutic strategies are desperately needed for both canines and humans.

Targeted radionuclide therapy (TRT) delivers cytocidal radiation in the form of α or β particles with high precision, reducing many of the side effects commonly associated with conventional external beam radiation [15,16]. Recently, the great therapeutic promise of TRT has been illustrated with the regulatory approvals of Xofigo^®^ (223Ra) for bone metastasis of prostate cancer [17,18] and Lutathera^®^ (177Lu-labeled peptide), a peptide receptor radionuclide therapy, for somatostatin receptor-positive gastroenteropancreatic neuroendocrine tumors (GEP-NETs) [19]. Lutathera^®^ was also evaluated for inoperable or metastatic paragangliomas and pheochromocytomas [20].

Radioimmunotherapy (RIT) is a subset of TRT where a radioisotope is bound to an antigen-specific antibody. With RIT, the cytotoxic radiation dose is delivered systemically to the targeted cells in a well-tolerated manner [21]. Compared to chemotherapy, RIT appears to be less affected by multidrug resistance mechanisms. Additionally, in contrast to naked antibody or T cell therapy, its action is independent of the host immune status or tumoral microenvironment [22].

The insulin-like growth factor receptor type 2 (IGF2R) sequesters the insulin-like growth factor (IGF-II) for internalization and degradation and also is considered a tumor suppressor with mutations being found in several cancers [23]. Unusually high levels of circulating IGF-II and simultaneous downregulation of IGF2R are correlated with the growth of human and murine tumors [24,25] while loss of function of IGF2R resulting in increased growth [26]. A recent publication has shown the consistent surface overexpression of IGF2R in human OS [27]. It was also previously found that a single nucleotide polymorphism (SNP) which alters methylation in an IGF2R CpG island was associated with an increased risk of OS development in humans [28]. Taken together, IGF2R expression appears to be both essential to and associated with the development of OS, making it an ideal therapeutic target. Sequence alignment of the IGFII binding region (Domains 11-FNII) in human, mouse, and dog have been demonstrated to be highly conserved (82% sequence identity) across these species [29].

Table 1 gives examples of the recent efforts to use TRT for treatment of OS in experimental models as well as advantages and disadvantages of different targets ad radionuclides [30,31,32,33,34]. Since the murine antibody 2G11 described for RIT of experimental OS in [ binds to human, murine, and canine IGF2R, we have chosen it as a reagent to perform immunohistochemistry (IHC) of canine OS tumors for the presence of IGF2R.

Dogs are known to be an excellent comparative model for different human cancers and particularly for OS. Primarily, OS incidence is 27 times higher in dogs than in humans, allowing for relatively frequent recruitment of canine patients with naturally occurring OS. Secondly, histologic architecture, biological behavior (such as bimodal age distribution, death caused by pulmonary metastasis, and genetic features), and current treatments are similar in both species. Finally, the compressed survival timeline in canine OS and the increased flexibility of veterinary research compared to its human counterpart accelerate the safety and efficacy evaluation of novel treatments [12,13]. Hence, dogs appear to be an ideal model for the evaluation of RIT against OS.

The primary objective of this study was to confirm the consistent cellular OS expression of IGF2R by using immunohistochemistry (IHC). The study aims to provide fundamental descriptive data for IGF2R expression for future evaluation of RIT in the treatment of canine and human OS and potentially other cancers. A secondary objective of this study was to investigate the prognostic value of IGF2R expression intensity in canine OS. This study is the first to demonstrate consistent expression of IGF2R in canine OS which will allow further investigation into the use of RIT targeting IFG2R for primary and metastatic OS in canines prior to clinical trials in humans with OS.

## 2. Results

### 2.1. Clinical and Epidemiological Data

A total of 34 canine OS samples were found available to include in this study. Samples consisted of 33 whole limbs and 1 biopsy (Jamshidi needle). Thirty-one out of the thirty-three whole limbs were from amputation, while the remaining two were from necropsy. Fourteen dogs (41%) were mixed breeds. Purebreds in the study population included Rottweilers (n = 7, 21%), German Shepherds (n = 2, 6%), Labrador retrievers (n = 2, 6%), and a single case (3%) each of the following breeds: Boxer, Bull Mastiff, Malamute, Goldendoodle, Golden retriever, Pit Bull, Great Dane, Greyhound, and Shetland Sheepdog. There were 19 spayed females (56%), 13 neutered males (38%), and 2 intact males (6%). The median age was 9 years (range 6–12). Median weight was 38.8 kg (range 10–62.6). The most common tumor location was proximal humerus (n = 10, 29%), followed by distal radius (n = 8, 24%), proximal femur (n = 4, 12%), distal femur (n = 4, 12%), distal tibia (n = 3, 9%), distal humerus (n = 2, 6%), and proximal tibia (3%), mid-humerus (3%) and scapula (3%) affecting one case each. Case descriptions are summarized in Table 1.

Table 2 summarizes signalment and tumor location of the 34 dogs affected with appendicular OS included in this study. Of all 34 dogs, 56% were female (n = 19) and 44% were male (n = 15). Data included in this table confirmed that all selected cases represent a group of dogs that correlates to what is observed clinically with most dogs affected by OS being a large breed dog (median weight 38.8 kg (range 10–62.6 kg)) of middle to geriatric age (median 9 years old (range 6–12)). The most commonly affected breeds were mixed breed dogs (n = 14, 41%) followed by Rottweilers (n = 7, 21%). The most common tumor location was the proximal humerus (n = 10, 29%) followed by the distal radius (n = 8, 24%).

### 2.2. Histopathology

Of the 34 cases, all samples were judged of adequate quality for IHC evaluation. All cases were confirmed to be OS. Osteoblastic OS was the most common subtype (30 dogs, 88%) of canine osteosarcoma, followed by the chondroblastic (3 dogs, 9%) and the fibroblastic (1 dog, 3%) subtypes (Table 3).

Table 3 summarizes histological subtype, treatment, survival time, status, and both visual intensity score (VIS) and corrected pixel density (CPD) scores of all 34 dogs included in the study. Osteoblastic OS was the most common histological subtype (30 dogs, 88%) of canine OS followed by the chondroblastic subtype (3 dogs, 9%). Nineteen dogs (56%) had intent to treat treatment (amputation or radiation therapy followed by chemotherapy) as their primary treatment. At the end of this study, 1 dog (3%) was lost to follow up, 28 dogs (82%) were dead, and 5 (15%) were alive. VIS was considered high in 24 cases (70.5%) and low in 10 cases (29.5%). Median CPD value was 54.94 (range 13.40–120.24). Cases with CPD equal or above the 75th percentile (70.75) were considered to have a high score.

### 2.3. IGF2R Immunostaining Intensity in Canine OS

**Figure 1:**
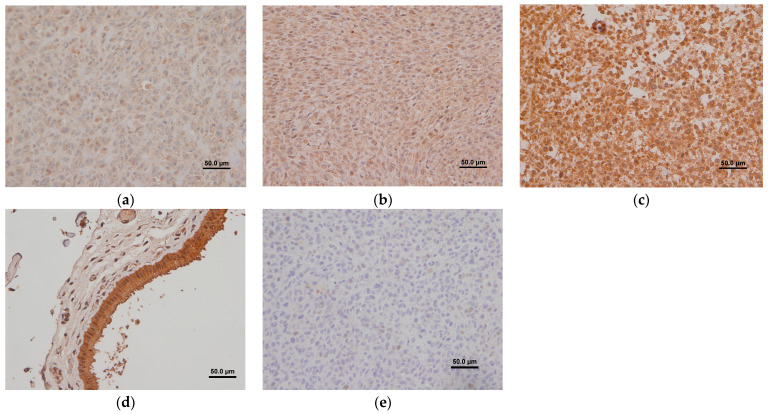
IHC of paraffin-embedded canine appendicular OS with 2G11 mAb: (**a**) representing a weak staining intensity; (**b**) representing a moderate staining intensity; (**c**) representing a strong staining intensity; (**d**) canine paraffin-embedded placenta was used as the positive control; (**e**) non-specific mAb MOPC-21 was used on paraffin-embedded canine appendicular OS as the negative control.

On visual assessment all samples stained positively to 2G11 mAb. Approximately 80–100% of the neoplastic cells were noted to have positive cytoplasmic staining in each respective sample. The intensity of staining in the majority of tumor cells in each lesion varied, however. Staining on visual assessment was considered strong, moderate, and weak in 24 (70.5%), 8 (23.5%) and 2 (6%) cases, respectively (Figure 1). Given the low number of weak staining samples, the VIS staining groups ‘moderate’ and ‘weak’ were combined (‘Low VIS’) for further analysis. This resulted in 24 dogs with High VIS and 10 dogs with Low VIS (Table 3).

**Figure 2:**
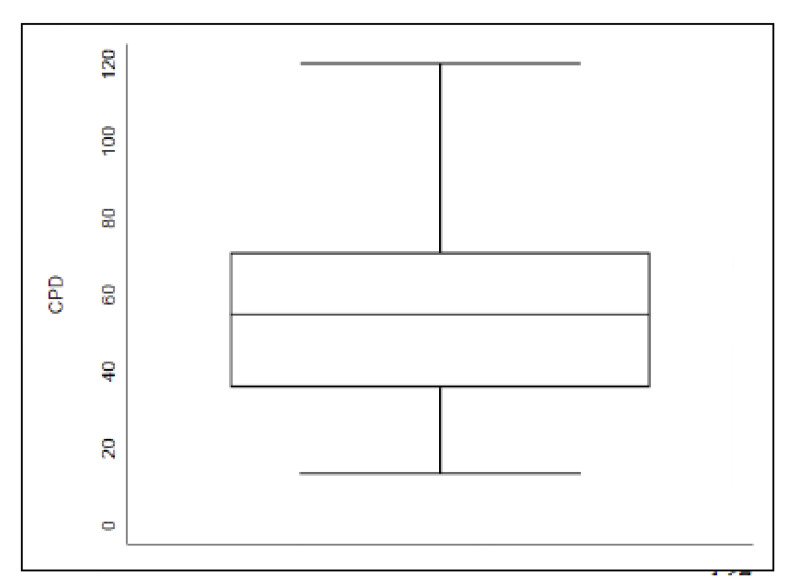
Whisker plot representing the CPD values for all dogs (n = 34). Median CPD value was 54.94 (25th percentile = 36.99; 75th percentile = 70.75). Higher CPD correlates with higher cellular IHC staining intensity of IGF2R.

**Figure 3:**
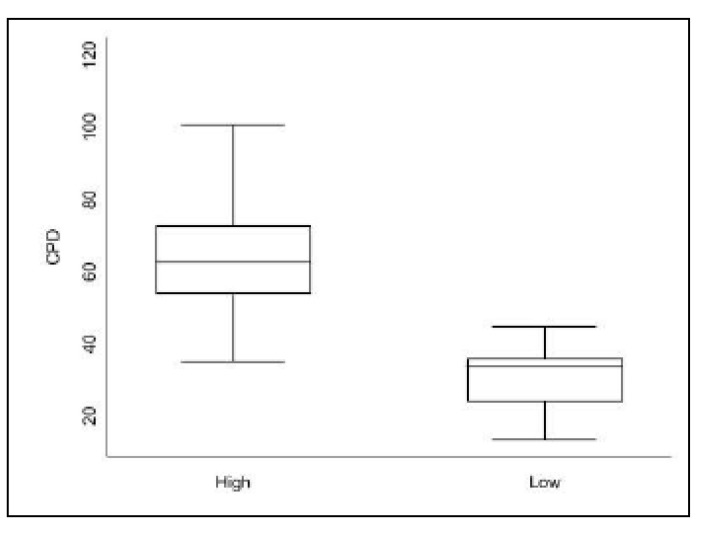
Whisker plot of CPD values of high (n = 25) vs. low (n = 9) VIS. High VIS CPD value (65.68) was significantly higher compared to low VIS CPD value (29.79) (*p* = 0.01).

The CPD values are represented in Figure 2. For all dogs, median CPD value was 54.94 (25th percentile = 36.99; 75th = 70.75). CPD was not correlated with age (*p* = 0.47). Samples in the high VIS group had a higher (65.68) CPD than samples in the low (29.79; *p* = 0.01) (Figure 3).

### 2.4. Flow Cytometry of Non-Neoplastic Canine Osteoblasts (CnOb) and Gracie (Canine OS) Cell Lines

**Figure 4:**
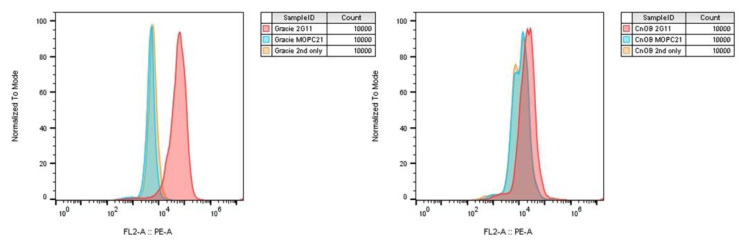
Flow cytometry histograms. **Left**: Gracie (canine OS cell line) binding intensity is significantly greater for 2G11 antibody (targeting IGF2R) compared to MOPC21 control and secondary antibody alone. **Right**: Non-neoplastic canine osteoblasts (CnOb cell line) have 2G11 binding comparable to MOPC21 control and secondary antibody alone. The findings indicate that neoplastic OS cells have increased IGF2R expression, where non-neoplastic osteoblasts do not have increased IGF2R expression.

Flow cytometric evaluation of Gracie (canine OS cell line) and non-neoplastic osteoblasts (CnOb) for 2G11 antibody, that targets cellular expression of IGF2R, suggest that neoplastic osteoblasts have significantly increased expression of IGF2R, while non-neoplastic osteoblasts do not have significantly increased IGF2R expression (see Figure 4). Data thus suggest that neoplastic canine osteoblasts, and not non-neoplastic canine osteoblasts, overexpress IGF2R, making this molecule a suitable target for IGF2R-mediated radioimmunotherapy.

### 2.5. Survival Analysis Related to Immunostaining Intensity of IGF2R in Canine OS

At the time of analysis, of the 34 dogs, 28 dogs were dead, 5 still alive and 1 was lost to follow up. The cause of death was suspected to be associated with progression of OS in 27 of 28 cases. The remaining case was euthanized due to coincidental development of uncontrolled glaucoma. Two dogs (6%) were euthanized upon diagnosis and 1 dog (3%) was lost to follow up, leaving 31 dogs (91%) available for survival analysis.

**Figure 5:**
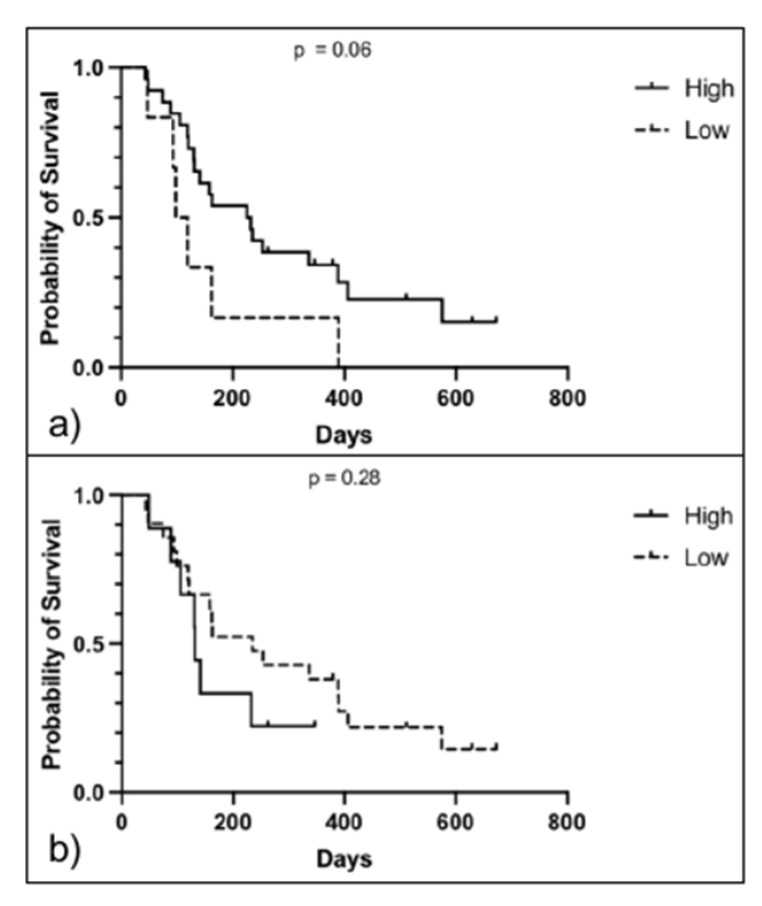
Kaplan–Meier survival analysis of all canine OS with high and low IFG2R expression. (**a**) Survival analysis of dogs with high versus low IGF2R expression on VIS. Survival time did not differ between high (229 days, 95% CI 85–524) and low (108 days, 95% CI 19–118) VIS (*p* = 0.06, Log Rank test; n = 25) (**b**) Survival analysis of dogs with high versus low IGF2R expression on CPD score. Survival time did not differ between high (131 days, 95% CI 23–134) and low (235 days, 95% CI 74–433) CPD score (*p* = 0.28, Log Rank test; n = 25).

There was no evidence of correlation for ST and CPD (linear regression; *p* = 0.42). Median ST was 163 days (95% confidence interval (CI) 120–336). Kaplan–Meier curves of high versus low VIS and CPD are represented in Figure 5. Log-rank (Mantle-Cox) test showed not statistical difference in the survival function between high (229 days) and low VIS (109 days; *p* = 0.06). There was no difference in the survival time between high (131 days) and low CPD samples (235 days; *p* = 0.28).

**Figure 6:**
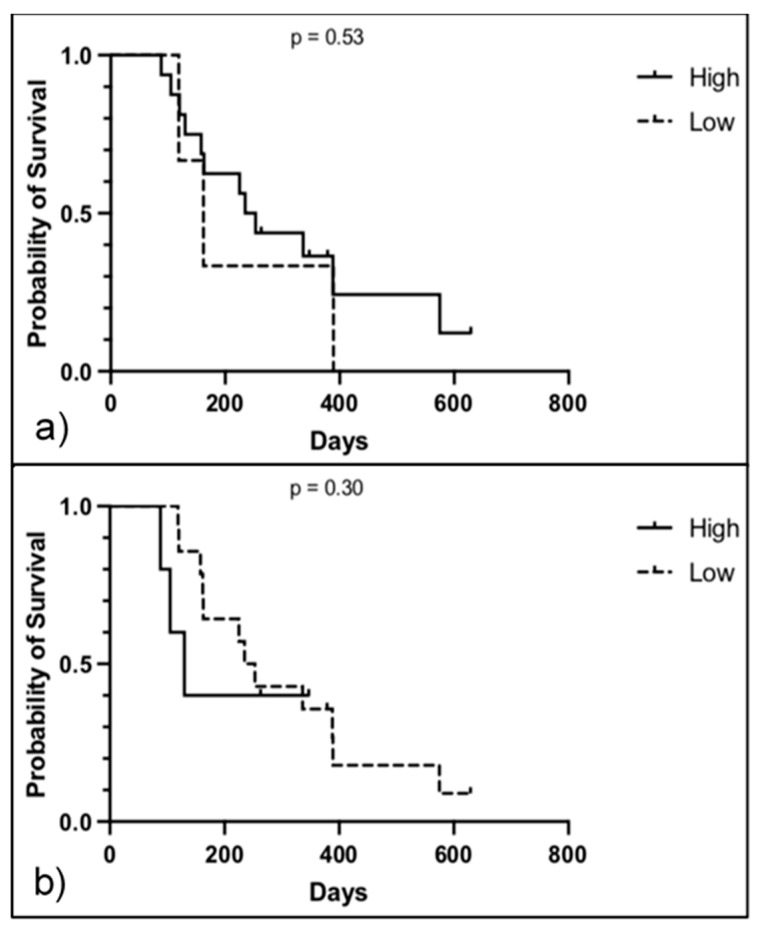
Kaplan–Meier survival analysis of the ITT subset with high and low IFG2R expression. (**a**) Survival analysis of dogs with high versus low IGF2R expression on VIS. Survival time did not differ between high (244 days, 95% CI 43–534) and low (162 days, 95% CI 19–235) VIS (*p* = 0.53, Log Rank test; n = 15) (**b**) Survival analysis of dogs with high versus low IGF2R expression on CPD score. Survival time did not differ between high (130 days, 95% CI 15–189) and low (244 days, 95% CI 53–665) CPD score (*p* = 0.58, Log Rank test; n = 15).

Further analysis was performed on the subset group of dogs categorized under ‘intent to treat’ (ITT). This group was deemed more homogenous and less prone to bias. From the 31 dogs included in the survival analysis, 19 (56%) of them received ITT. Three dogs were still alive at the time of analysis, and one was euthanized due to uncontrolled glaucoma. Linear regression between the ST and CPD showed no correlation with ST (*p* = 0.25). Kaplan–Meier curves of high versus low VIS and CPD are represented in Figure 6. Log-rank (Mantle-Cox) test showed not statistical difference in the survival function between high (244 days) and low VIS (162 days; *p* = 0.53). There was no difference in the survival time between high (130 days) and low CPD samples (244 days; *p* = 0.30).

## 3. Discussion

Based on this study, we concluded that the primary aim could be answered by confirming the consistent and homogeneous expression of IGF2R in canine appendicular OS cells. The semi-quantitative grading system proposed by Scandalis and Damanesco could not be applied to neoplastic cell populations in our study, as all samples showed a percentage of cell positivity to 2G11 close to 100%. Following this finding, OS samples were classified based on their staining intensity. On VIS, most samples (70.5%) were staining strongly to 2G11 mAb. While results of this study showed that CPD values of low versus high VIS samples were significantly different, the two methods of assessing the IGF2R staining intensity were not considered to be comparable. Furthermore, the results of flow cytometry for IGF2R expression on cultured non-neoplastic canine osteoblasts and cultured canine OS cells (Gracie) showed overexpression of IGF2R on only the latter. This suggests the potential for radioimmunotherapy targeting IGF2R to be selective for neoplastic osteoblasts.

Only two major negative prognostic factors, increased serum levels of alkaline phosphatase (SALP) and tumor location, have been clearly illustrated in canine OS [35,36,37]. It is known that a subset of OS will have a prolonged survival in dogs. Currently there is no known method to identify these cases prior to treatment in the veterinary field. Being able to correctly identify this subset of canine appendicular OS would be advantageous for both clinician and pet owners. A recent retrospective study suggests that SUVmax measured by an 18F-FDG PET/CT was significantly prognostic for median survival time (SUVmax of ≥7.4; 254 days, SUVmax of <7.4; 680 days) [38]. However, the proposed cut-off cannot be translated to 18F-FDG PET/CT data obtained at other institutions. Low expression of the parathyroid hormone receptor 1 (PTHR1) was also recently associated with an improved outcome [39]. Moreover, a recent study showed that expression of IGF1R in canine osteosarcoma is associated with a poorer prognosis [40]. Therefore, evaluating if IGF2R expression was correlated with survival was deemed to be an important second aim of this study. Results however showed that the magnitude of IGF2R expression was not of significant prognostic value. Failure to detect a significant difference in ST for dogs with high and low CPD could be of a type 2 error due to the small sample size of this study and lack of power. Additionally, the method to assess the CPD was evaluating the mean of 30 manually selected cells per sample. The authors of this study cannot verify if using an automatic system that would evaluate all cells for each sample would have resulted in different conclusions. Prospective clinical trial with a larger population of dogs would be required to confirm the findings of this study.

The most common histological subtype identified in this study was the osteoblastic form (n = 30), consistent with previous studies. Osteoblastic is also the most common subtype in human OS, emphasizing the similarities of this disease in both species [14]. The histologic subtype in canine appendicular OS was not previously associated with different outcome [41]. However, a recent study showed that patients with fibroblastic osteosarcoma had a significantly improved overall survival compared to those with osteoblastic and chondroblastic subtypes, the latter two carrying the worse prognosis [42].

Previously approved treatments with RIT in humans are known to be well tolerated despite the presence of the target in normal tissue. This is illustrated by the tolerability of Lutathera^®^, where somatostatin receptors have been described in normal neurological, lymphoid, intestinal, and endocrine tissues [43]. Therefore, the authors of this study do not consider the presence of IGF2R in normal tissue to preclude the use of RIT in canine OS. The safety of this treatment, despite the presence of the target in normal tissue, is believed to derive from the inherent increased tolerability and repair to radiation damage of normal tissue compared to tumors. Based on previous work done by Dadachova et al., RIT targeting 2G11 in a xenograph murine model was well tolerated. As the mice were euthanized after 12 days of observation, only acute side effects could be assessed. Therefore, potential late complications of this treatment are still to be evaluated. To further assess the biodistribution and potential risk of using RIT in dogs, gamma-emitting studies on a cohort of healthy research dogs is recommended prior to clinical trials on client owned dogs.

While most canine samples stained moderately or strongly to 2G11 (32/34), variability in antigen density could be appreciated. This could raise the concern of a potential diminished response (to applied TRT) for the cases that has a lower density of IGF2R in OS cells. Previous work with murine species using microSPECT/CT showed a high IGF2R-specific uptake of 111In-2G11 in tumors that was not dependent on the in vitro levels of IGF2R expression [34]. Therefore, current available data expect all canine patients to similarly uptake the radio-labeled mAb. Receptor density is also not expected to affect efficacy of treatment especially if an α-emitter is used as α particle radiation’s weight factor is 20 (1 for β rays) and one or two hits are theoretically enough to cause cell death.

Future directions aim to utilize a humanized version of the 2g11 mAb to assess the efficacy of RIT targeting IGF2R in canine patients. The use of cross species mAb is known to be well tolerated, as illustrated by the approval of Zevalin^®^ and Bexxar^®^, both murine radiolabeled mAb targeting CD20 for the treatment of Non-Hodgkin’s Lymphoma in human [44]. Therefore, the use of a humanized mAb should be efficient and safe to use in canine patients.

Limitations of this study are inherent to its retrospective nature like the heterogenicity of treatment received and follow up information. Another limitation of this study is that IGF2R expression of canine appendicular OS metastasis was not assessed. It is known that metastatic disease is often more anaplastic compared to the primary tumor. One could speculate that decreased differentiation could lead to a decreased expression or inexpression of IGF2R. As demonstrated, decreased IGF2R expression does not appear to be a major concern to predict efficiency of RIT, but expression of IGF2R in canine OS metastasis remain to be confirmed.

## 4. Materials and Methods

**Figure 7:**
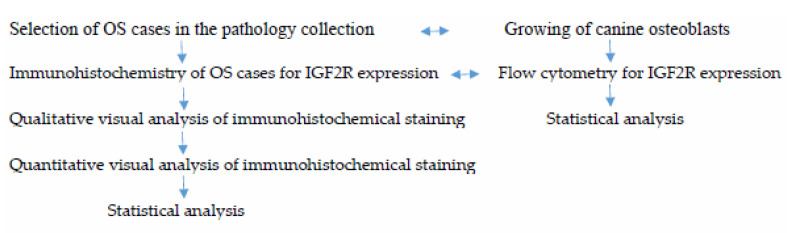
Workflow of the experiments. The IHC analysis of the clinical cases and the growth and flow cytometry of canine osteoblasts were conducted in parallel.

Figure 7 shows the workflow of the experiments described in the materials and methods below.

### 4.1. Case Selection

Medical records of dogs presented to the Western College of Veterinary Medicine from the period of January 2014 to January 2019 diagnosed with appendicular OS through Prairie Diagnostic Services (PDS) were reviewed. Information regarding case signalment (age, gender, breed, weight), clinical signs, tumor location, staging, treatment received, survival time and cause of death were collected from patient’s medical records. Referred veterinarians were contacted by phone or email to retrieve information missing from the available medical records. Tumor samples procured via biopsy, limb amputation, or necropsy from the PDS archive were included in the study. All samples were reviewed (RD) to assess for sample quality, to confirm the diagnosis of OS and its subtype. Cases were excluded if the sample quality was deemed poor, sample size was too small for IHC or if review of the tumor architecture resulted in omission of OS as a definitive diagnosis.

### 4.2. Immunohistochemistry

Immunohistochemical staining (IHC) was performed with IGF2R antibodies on all cases (n = 34). OS canine paraffin wax-embedded, decalcified tissue sections (3 μm) were created using a microtome and fixed on positively charged microscope slides (Superfrost Plus, Thermo Fisher, Waltham, MA, USA). Sections were deparaffinized using xylene and graded alcohol treatment. Endogenous peroxidases were inactivated using a methanol H_2_O_2_ solution prior to heat induced antigen retrieval in tris-EDTA pH 9 buffer for 20 min at 97 °C. Immunohistochemical staining for detection of IGF2R expression in canine OS was performed at PDS using an automated staining platform (Autostainer Plus, Dako Canada Inc., Mississauga, ON, Canada). Heat-induced epitope retrieval was performed, and the primary murine anti-IGF2R antibody (2G11) was applied for 30 min at a 1:100 dilution. Binding was detected using an HRP-labeled polymer detection reagent (Dako Canada Inc., Mississauga, ON). The staining was visualized using 3,3′-diaminobenzidine tetrahydrochloride (DAB) (Dako Canada Inc., Mississauga, ON, Canada) as the chromogen. Using the same IHC protocol, MOPC-21 IgG1 was used instead of the primary antibody as the isotype negative control. Canine paraffin-embedded placenta was used as the positive control. ProbeOn Plus charged slides (Thermo Fisher, Waltham, MA, USA) were used for canine placenta positive control.

### 4.3. Qualitative Visual Analysis of Immunohistochemical Staining

A subjective method to assess staining intensity for IGF2R expression was performed by directly evaluating the stained slide, named as Visual Intensity Score (VIS). Original visual assessment was planned to be based on the semiquantitative scoring system proposed by Skandalis et al. and Damasceno et al. [45,46], which includes the overall percentage of positively stained tissue (0–100%) and staining intensity. Samples were evaluated by the resident (CB) and clinical pathologist (RD) at their most representative area at 400× magnification. The slides were considered positive if any staining was observed and negative if no staining was observed. After first evaluation of the immunohistochemical staining, it was noticed that nearly all OSA cells were positive for IGF2R. Related to this finding, the previously proposed semi-quantitative grading scheme could not be established. A Visual Intensity Score (VIS) was therefore based on the staining intensity only. “Strong staining” was represented by high intensity staining diffusely within the cytoplasm of most tumor cells; “moderate staining” was represented by moderate intensity staining coupled with localized or diffuse staining in the cytoplasm of tumor cells, while “weak staining” was represented by pale (yet convincingly positive) cytoplasmic staining that was more often focal within the cytoplasm of tumor cells. For further analysis, slides with strong staining were considered to have a high VIS and slides with moderate and weak staining were considered to have low VIS.

### 4.4. Quantitative Analysis of Immunohistochemical Staining

Quantitative analysis of IGF2R expression staining intensity was performed using Image-Pro Premier Version 9.3.3 software, that assesses the pixel density of the immunostaining. IHC images were acquired on Olympus Bx41 microscope. A total of 30 OS cells were manually selected for each case, using the same representative area used in the VIS method. As the nucleus was noted to be significantly darker than the cytoplasm and that visualization of the nucleus was not homogeneous amongst cases, only pixel density of the cytoplasm was performed to avoid bias. The area of the cytoplasm for which pixel density was to be assessed was outlined (CB) using the software. Pixel density is calculated by the quantity of light reaching the camera objective. In that perspective, pixel score was inversely correlated to the immunostaining intensity. For a practical standpoint, raw pixel score data were adjust by the formula “Corrected Pixel Density” = Negative point—“Raw Pixel Density”, referred to as CPD for this study. Therefore, higher CPD correlates with higher cellular IHC staining for IGF2R. Calibration was performed by selecting a negative point (background not containing staining, 196.11) and a positive point (cytoplasm of a visually strong intensity, 73.36). The same calibration was then used for all cases. Preliminary analysis of CPD values showed a highly skewed distribution with most values close to the median. Therefore, the 75th percentiles value was used as a cut-off to create categories for high and low CPD, with CPD values of slides scoring ≥ 75th percentile were considered to have high CPD value, while slides scoring < 75th percentiles were considered to have low CPD value.

### 4.5. Cell Lines

Canine osteoblast cell line (CnOb) was obtained from Sigma-Aldrich (CN406-05) and canine patient derived osteosarcoma (Gracie) cell line was a kind gift from Dr Doug Thamm’s laboratory at Colorado State University’s School of Veterinary Medicine. The CnOb cells were cultured in canine osteoblast growth medium (Cn417-500). Gracie cell line was cultured in RPMI-1640 medium supplemented with 10% FBS, 1% sodium pyruvate, 1% non-essential amino acids, and 100 U penicillin/0.1 mg/mL streptomycin.

### 4.6. Flow Cytometry

A 96-well plate consisting of 150,000 cells in each well was incubated with 300 nM concentration of the primary antibody 2G11 and Mouse IgG1 isotype control MOPC-21 for 30 min. Cells were then washed thrice with FACS buffer (PBS + 0.5% BSA or 2% serum + 0.02% azide). Goat anti-mouse IgG2a H&L, PE from Abcam (ab74490) was used as secondary antibody for 2G11 and rat anti-mouse IgG1, PE, eBioscience^TM^ (12-4015-82) was used for MOPC-21. The cells were then incubated for 30 min and washed again three times with FACS buffer before reading in the CytoFlex machine (Beckman Coulter, Indianapolis, USA). The data was analyzed using FlowJo.

### 4.7. Statistical Analysis

Basic descriptive statistics were used to assess the agreement between the two tests (VIS and CPD) for evaluating the staining intensity for IGF2R expression. Continuous variables were assessed for normality using the Shapiro–Wilk test. Student t-test was used to assess difference between CPD of slides that scored high vs. low on VIS. Correlation between pixel density and age was assessed with a linear regression test.

Cases were excluded from survival analysis if they were euthanized at diagnosis or if no data was available for longer than 60 days post diagnosis (early loss to follow up). Dogs that were still alive or died due to a reason unrelated to OS were censored. Dogs for which the reason of death was unknown were considered to have died from the OS. Survival time (ST) was defined as the number of days between the diagnosis and the time of death. Correlation between CPD and ST was assessed using linear regression. Kaplan–Meier analysis was used to investigate ST of high versus low staining intensity for both VIS and CPD methods. Difference in ST between high and low staining intensity was assessed with the log-rank test.

To avoid possible bias due to the heterogeneity of treatments, further analysis was performed on a subset group including exclusively dogs who received treatments in intent to treat (ITT). ITT was defined as dogs that had either amputation or SRT treatment for local treatment followed by at least 1 dose of carboplatin.

A *p* value of ≤0.05 was considered significant, and 95% confidence intervals were used. Stata version 15.1 was used for the descriptive data and Prism6 GraphPad was used for the survival analysis.

## 5. Conclusions

In conclusion, this study demonstrated the consistent IHC expression of IGF2R for the first time in spontaneously occurring canine appendicular OS, confirming that dogs are an excellent comparative model for humans in the development of RIT targeting this receptor. The intensity of IGF2R overexpression alone was not found to be of prognostic significance in this study, however, demonstration of IGF2R overexpression in canine OS cells establishes proof of concept that this molecule is a suitable target for further investigation of RIT for treatment of primary and metastatic OS, or potentially other cancers that may overexpress IGF2R. This information helps set the stage for clinical trials of RIT targeting IGF2R for primary and metastatic OS in canines prior to clinical trials in humans with OS.

## Figures and Tables

**Table 1:** Examples of TRT use for treatment of OS in experimental models.

Targeting Molecule	Radionuclide	Advantages	Disadvantages	Reference
EDTMP	^153^Sm	Ease of synthesis and administration	Lack of targeting soft tissue metastasis	[30]
Polymeric Phosphonates	^153^Sm	Ease of synthesis and administration	Non-specific targeting of non-osseous tumors based on enhanced permeability and retention (the EPR effect)	[31]
Antibody to CD146	^177^Lu	Antigen-specific delivery of the radionuclide to the tumor	Bone marrow as a possible dose-limiting organ	[32]
Antibody to human IGF2R	^188^Re	Antigen-specific delivery of the radionuclide to the tumor	Antibody binds only to human IGF2R making it difficult to assess toxicity in animal models	[22]
Antibody to major histocompatibility complex class I chain-related protein A and B	^211^At	Antigen-specific delivery of the radionuclide to the tumor	Availability of ^211^At is limited to only a few places around the world	[33]
Antibody to human, murine, and canine IGF2R	^177^Lu	Antigen-specific delivery of the radionuclide to the tumor; possible to use for toxicity evaluation in rodents and dogs	The antibody is murine which precludes its repeated administrations to humans	[34]

**Table 2:** Summary table including signalment (breed, sex, neuter status, age) and tumor location of 34 dogs with appendicular OS; FS, female spayed; F, Female intact; MC, Male castrated; M, Male intact.

#	Breed	Sex	Age	Location
1	Labrador Retriever Cross	FS	10	Distal radius
2	German Shepherd Cross	FS	9	Proximal humerus
3	Boxer Cross	MC	10	Femoral head
4	Boxer	FS	9.5	Distal humerus
5	Rottweiler	MC	10	Proximal tibia
6	Rottweiler	MC	9	Distal tibia
7	Labrador Retriever Cross	FS	7	Distal femur
8	German Shepherd	MC	9	Distal radius
9	Rottweiler	FS	10	Proximal femur
10	Great Dane	FS	7	Distal radius
11	Rottweiler Cross	FS	9	Proximal humerus
12	Rottweiler	FS	9	Distal radius
13	Bull Mastiff	MC	9	Proximal humerus
14	Boxer Cross	FS	6	Scapula
15	Doberman Cross	MC	7	Proximal humerus
16	Border Collie Cross	MC	9	Distal radius
17	Rottweiler	MC	8	Mid humerus
18	Rottweiler	FS	9	Distal femur
19	Pitbull	FS	8	Proximal humerus
20	Goldendoodle	FS	8.5	Proximal humerus
21	Giant Schnauzer Cross	FS	6	Distal tibia
22	Rottweiler Cross	MC	9	Distal femur
23	Shetland Sheepdog	M	12	Proximal humerus
24	Labrador Retriever	FS	7.5	Proximal humerus
25	Golden Retriever	MC	8	Proximal humerus
26	Labrador Retriever Cross	FS	11	Proximal humerus
27	Belgian Malinois Cross	MC	7	Distal tibia
28	Greyhound	MC	7.5	Distal radius
29	Rottweiler	M	8	Proximal femur
30	Rottweiler Cross	FS	10	Distal femur
31	Labrador Retriever	FS	10	Distal humerus
32	German Shepherd	FS	11	Proximal humerus
33	Malamute	MC	8	Distal radius
34	Rottweiler	FS	6.5	Proximal humerus

**Table 3:** 2. Summary table including histologic subtype, treatment, survival time, status and both VIS and CPD score; Ob, osteoblastic; Ch, Chondroblastic; Fb, Fibroblastic; Amp, Amputation; SRT, Stereotactic radiation therapy; RT, Radiation therapy; CHOP, Madison-Wisconsin Protocol; BPN, Bisphosphonates; NA, Not available; LFU, lost to follow up. The number in parenthesis represents the number of carboplatin (chemotherapy) doses received.

	OS Subtype	Primary Treatment	ST (Days)	Status	Visual Intensity Score	Corrected Pixel Density	Corrected Pixel Density Score
1	Ob OSA	Amp	43	dead	High	69.91	High
2	Ob OSA	Amp + Carboplatin (6)	235	dead	High	54.97	High
3	Ob OSA	Amp	NA	LFU	Low	44.62	Low
4	Ob OSA	Amp	47	dead	Low	33.67	Low
5	Ob OSA	Amp + Carboplatin (2)	163	dead	High	54.92	Low
6	Ob OSA	Amp	131	dead	High	71.03	High
7	Ob OSA	Amp + Carboplatin (3)	575	dead	High	58.18	High
8	Ob OSA	Amp + Carboplatin (6)	388	dead	High	35.13	Low
9	Ob OSA	Amp + Carboplatin (2)	105	dead	High	100.89	High
10	Ob OSA	Amp + palladia	93	dead	Low	40.14	Low
11	Ob OSA	SRT + Carboplatin (6)	379	dead	High	59.55	High
12	Ob OSA	Amp + Carboplatin (6)	253	dead	High	42.57	Low
13	Ob OSA	Palliative	74	dead	High	50.96	Low
14	Ch OSA	Amp	48	dead	High	120.24	High
15	Ob OSA	Amp + Carboplatin (4)	119	dead	Low	23.92	Low
16	Ob OSA	Amp	232	dead	High	72.73	High
17	Ob OSA	Amp	406	dead	Low	35.94	Low
18	Ob OSA	Amp	141	dead	High	79.78	High
19	Ob OSA	Amp	672	dead	High	21.65	Low
20	Fb OSA	Amp + Carboplatin (6)	162	dead	Low	24.55	Low
21	Ob OSA	Amp + Carboplatin (2)	88	dead	High	93.68	High
22	Ch OSA	Amp + Carboplatin (6)	336	dead	High	62.52	High
23	Ob OSA	Amp + Carboplatin (3)	347	alive	High	71.82	High
24	Ob OSA	Amp + CHOP	98	dead	Low	17.55	Low
25	Ob OSA	Amp + Carboplatin (6)	389	dead	Low	34.34	Low
26	Ob OSA	Amp + Carboplatin (3)	130	dead	High	112.58	High
27	Ob OSA	Amp + Carboplatin (6)	629	alive	High	41.28	Low
28	Ch OSA	BPN + palliative RT + Carbo (6)	511	alive	High	64.92	High
29	Ob OSA	Euthanasia	0	dead	Low	13.40	Low
30	Ob OSA	Euthanasia	0	dead	High	65.77	High
31	Ob OSA	Amp + Carboplatin (6)	120	dead	High	57.58	High
32	Ob OSA	Amp + Carboplatin (1)	158	dead	High	53.74	Low
33	Ob OSA	Amp + Carboplatin (1)	225	dead	High	52.99	Low
34	Ob OSA	Amp + Carboplatin (4)	263	alive	High	72.69	High

## Data Availability

The data presented in this study are available in this article.

## References

[1] Ehrhart N.P., Neil I.C., Fan T.M., Withrow S.J., Vail D.M. (2020). Tumors of the skeletal system. Withrow and MacEwen’s Small Animal Clinical Oncology.

[2] Liptak J.M., Dernell W.S., Ehrhart N.P., Withrow S.J. (2004). Canine appendicular osteosarcoma: Diagnosis and palliative treatment. Compend. Contin. Educ. Vet..

[3] Mirabello L., Troisi R.J., Savage S.A. (2009). Osteosarcoma incidence and survival rates from 1973 to 2004: Data from the surveillance, epidemiology, and end results program. Cancer.

[4] Spodnick G.J., Berg J., Rand W.M., Schelling S.H., Couto G., Harvey H.J., Henderson R.A., MacEwen G., Mauldin N., McCaw D.L. (1992). Prognosis for dogs with appendicular osteosarcoma treated by amputation alone: 162 cases (1978–1988). J. Am. Vet. Med. Assoc..

[5] Bergman P.J., MacEwen E.G., Kurzman I.D., Henry C.J., Hammer A.S., Knapp D.W., Hale A., Kruth S.A., Klein M.K., Klausner J. (1996). Amputation and Carboplatin for Treatment of Dogs with Osteosarcoma: 48 Cases (1991 to 1993). J. Vet. Intern. Med..

[6] Phillips B., Powers B.E., Dernell W.S., Straw R.C., Khanna C., Hogge G.S., Vail D.M. (2009). Use of single-agent carboplatin as adjuvant or neoadjuvant therapy in conjunction with amputation for appendicular osteosarcoma in dogs. J. Am. Anim. Hosp. Assoc..

[7] Saam D.E., Liptak J.M., Stalker M.J., Chun R. (2011). Predictors of outcome in dogs treated with adjuvant carboplatin for appendicular osteosarcoma: 65 cases (1996–2006). J. Am. Vet. Med. Assoc..

[8] Farese J.P., Milner R., Thompson M.S., Lester N., Cooke K., Fox L., Hester J., Bova F.J. (2004). Stereotactic radiosurgery for treatment of osteosarcomas involving the distal portions of the limbs in dogs. J. Am. Vet. Med. Assoc..

[9] Covey J.L., Farese J.P., Bacon N., Schallberger S.P., Amsellem P., Cavanaugh R., Milner R.J. (2014). Stereotactic Radiosurgery and Fracture Fixation in 6 Dogs with Appendicular Osteosarcoma. Vet. Surg..

[10] Boston S.E., Vinayak A., Lu X., LaRue S., Bacon N., Bleedorn J.A., Souza C.H.M., Ehrhart N.P. (2017). Outcome and complications in dogs with appendicular primary bone tumors treated with stereotactic radiotherapy and concurrent surgical stabilization. Vet. Surg..

[11] Kim C., Matsuyama A., Mutsaers A.J., Woods J.P. (2017). Retrospective evaluation of toceranib (Palladia) treatment for canine metastatic appendicular osteosarcoma. Can. Vet. J..

[12] Morello E., Martano M., Buracco P. (2011). Biology, diagnosis and treatment of canine appendicular osteosarcoma: Similarities and differences with human osteosarcoma. Vet. J..

[13] Rowell J.L., McCarthy D.O., Alvarez C.E. (2011). Dog models of naturally occurring cancer. Trends Mol. Med..

[14] Simpson S., Dunning M.D., de Brot S., Grau-Roma L., Mongan N.P., Rutland C.S. (2017). Comparative review of human and canine osteosarcoma: Morphology, epidemiology, prognosis, treatment and genetics. Acta Vet. Scand..

[15] Milenic D.E., Brady E.D., Brechbiel M.W. (2004). Antibody-targeted radiation cancer therapy. Nat. Rev. Drug Discov..

[16] Larson S.M., Carrasquillo J.A., Cheung N.K., Press O.W. (2015). Radioimmunotherapy of human tumors. Nat. Rev. Cancer.

[17] Sartor O., Heinrich D., Mariados N., Méndez Vidal M.J., Keizman D., Thellenberg Karlsson C., Peer A., Procopio G., Frank S.J., Pulkkanen K. (2019). Re-treatment with radium-223: 2-year follow-up from an international, open-label, phase ½ study in patients with castration-resistant prostate cancer and bone metastases. Prostate.

[18] (2013). Xofigo (radiumRa223dichloride) Injection for Intravenous Use [Package Insert].

[19] Alex N.E., Christoph B. (2005). Does 177Lu-labeled octreotate improve the rate of remission of endocrine gastroenteropancreatic tumors?. Nat. Clin. Pract. Endocrinol. Metab..

[20] Zandee W.T., Feelders R.A., Duijzentkunst D.A.S., Hofland J., Metselaar R.M., Oldenburg R.A., van Linge A., Kam B.L.R., Teunissen J.J.M., Korpershoek E. (2019). Treatment of inoperable or metastatic paragangliomas and pheochromocytomas with peptide receptor radionuclide therapy using 177Lu-DOTATATE. Eur. J. Endocrinol..

[21] Sharkey R.M., Goldenberg D.M. (2005). Perspectives on cancer therapy with radiolabeled monoclonal antibodies. J. Nucl. Med..

[22] Geller D.S., Morris J., Revskaya E., Kahn M., Zhang W., Piperdi S., Park A., Koirala P., Guzik H., Hall C. (2016). Targeted therapy of osteosarcoma with radiolabeled monoclonal antibody to an insulin-like growth factor-2 receptor (IGF2R). Nucl. Med. Biol..

[23] Falls J.G., Pulford D.J., Wylie A.A., Jirtle R.L. (1999). Genomic imprinting: Implications for human disease. Am. J. Pathol..

[24] Toretsky J.A., Helman L.J. (1996). Involvement of IGF-II in human cancer. J. Endocrinol..

[25] Hassan A.B., Howell J.A. (2000). Insulin-like growth factor II supply modifies growth of intestinal adenoma in Apc(Min/+) mice. Cancer Res..

[26] Foulstone E., Prince S., Zaccheo O., Burns J.L., Harper J., Jacobs C., Church D., Hassan A.B. (2005). Insulin-like growth factor ligands, receptors, and binding proteins in cancer. J. Pathol..

[27] Hassan S.E., Ba M.B., Kim M.Y., Lin J., Bs S.P., Gorlick R., Geller D.S. (2012). Cell surface receptor expression patterns in osteosarcoma. Cancer.

[28] Savage S.A., Woodson K., Walk E., Modi W., Liao J., Douglass C., Hoover R.N., Chanock S.J., National Osteosarcoma Etiology Study Group (2007). National Osteosarcoma Etiology Study Group. Analysis of genes critical for growth regulation identifies Insulin- like Growth Factor 2 Receptor variations with possible functional significance as risk factors for osteosarcoma. Cancer Epidemiol. Biomark. Prev..

[29] Brown J., Delaine C., Zaccheo O.J., Siebold C., Gilbert R., Van Boxel G., Denley A., Wallace J.C., Hassan A., Forbes B. (2008). Structure and functional analysis of the IGF-II/IGF2R interaction. EMBO J..

[30] Burland O.S., Skretting A., Solheim O.P., Aas M. (1996). Targeted radiotherapy of osteosarcoma using 153SM-Edtmp: A new promising approach. Acta Oncol..

[31] Popwell S.J., Schulz M.D., Wagener K.B., Batich C.D., Milner R. J., Lagmay J., Bolch W.E. (2014). Synthesis of polymeric phosphonates for selective delivery of radionuclides to osteosarcoma. Cancer Biother. Radiopharm..

[32] Westrom S., Bondsdorff T.B., Abbas N., Bruland O.S., Jonasdottir T.J., Maelandsmo G.M., Larsen R.H. (2016). Evaluation of CD146 as target for radioimmunotherapy against osteosarcoma. PLoS ONE.

[33] Li H.K., Hasegawa S., Nakajima J.I., Morokoshi Y., Miengishi K., Nagatsu K. (2018). Targeted cancer cell ablation in mice by an α particle emitting astatine-211-labelled antibody again major histocompatibility complex class I chain-related protein A and B. Biochem. Biophys. Res. Commun..

[34] Karkare S., Allen K.J.H., Jiao R., Malo M.E., Dawicki W., Helal M., Godson D.L., Dickinson R., MacDonald-Dickinson V., Yang R. (2019). Detection and targeting insulin growth factor receptor type 2 (IGF2R) in osteosarcoma PDX in mouse models and in canine osteosarcoma tumors. Sci. Rep..

[35] Boerman I., Selvarajah G.T., Nielen M., Kirpensteijn J. (2012). Prognostic factors in canine appendicular osteosarcoma—A meta-analysis. BMC Vet. Res..

[36] Garzotto C.K., Berg J., Hoffmann W.E., Rand W.M. (2000). Prognostic significance of serum alkaline phosphatase activity in canine appendicular osteosarcoma. J. Vet. Intern. Med..

[37] Ren H.-Y., Sun L.-L., Li H.-Y., Ye Z.-M. (2015). Prognostic Significance of Serum Alkaline Phosphatase Level in Osteosarcoma: A Meta-Analysis of Published Data. BioMed Res. Int..

[38] Griffin L.R., Thamm D.H., Brody A., Selmic L.E. (2019). Prognostic value of fluorine18 flourodeoxyglucose positron emission tomography/computed tomography in dogs with appendicular osteosarcoma. J. Vet. Int. Med..

[39] Al-Khan A.A., Nimmo J.S., Tayebi M., Ryan S.D., Simcock J.O., Tarzi R., Kuntz C.A., Saad E.S., Day M.J., Richardson S.J. (2020). Parathyroid hormone receptor 1 (PTHR1) is a prognostic indicator in canine osteosarcoma. Sci. Rep..

[40] Maniscalco L., Iussich S., Morello E., Martano M., Gattino F., Miretti S., Biolatti B., Accornero P., Martignani E., Sánchez-Céspedes R. (2015). Increased expression of insulin-like growth factor-1 receptor is correlated with worse survival in canine appendicular osteosarcoma. Vet. J..

[41] Schott C.R., Tatiersky L.J., Foster R.A., Wood G.A. (2018). Histologic Grade Does Not Predict Outcome in Dogs with Appendicular Osteosarcoma Receiving the Standard of Care. Vet. Pathol..

[42] Al-Khan A., Nimmo J., Day M., Tayebi M., Ryan S., Kuntz C., Simcock J., Tarzi R., Saad E., Richardson S. (2020). Fibroblastic Subtype has a Favourable Prognosis in Appendicular Osteosarcoma of Dogs. J. Comp. Pathol..

[43] Reubi J.C., Schaer J.C., Markwalder R., Waser B., Horisberger U., Laissue J. (1997). Distribution of somatostatin receptors in normal and neoplastic human tissues: Recent advances and potential relevance. Yale J. Biol. Med..

[44] Sachpekidis C., Jackson D.B., Soldatos T.G. (2019). Radioimmunotherapy in Non-Hodgkin’s Lymphoma: Retrospective Adverse Event Profiling of Zevalin and Bexxar. Pharmaceuticals.

[45] Skandalis S.S., Labropoulou V.T., Ravazoula P., Likaki-Karatza E., Dobra K., Kalofonos H.P., Karamanos N.K., Theocharis A.D. (2011). Versican but not decorin accumulation is related to malignancy in mammographically detected high density and malignant appearing microcalcifications in non-palpable breast carcinomas. BMC Cancer.

[46] Damasceno K.A., Ferreira E., Estrela-Lima A., Gamba Cde O., Miranda F.F., Alves M.R., Rocha R.M., de Barros A.L., Cassali G.D. (2016). HER-2 and EGFR mRNA Expression and Its Relationship with Versican in Malignant Matrix-Producing Tumors of the Canine Mammary Gland. PLoS ONE.

